# Differences in physiological response to an oral glucose tolerance test in adult Maasai men and women

**DOI:** 10.1007/s00592-026-02710-8

**Published:** 2026-05-22

**Authors:** Dirk Lund Christensen, Cathrine Olesen Emborg, Kaushik Laxmidas Ramaiya, Venance Philip Maro, Ib Christian Bygbjerg, Joseph Sironga, Jens Juul Holst, Kajiru Kilonzo, Bolette Hartmann, Flemming Dela, Steen Larsen, Jørn Wulff Helge

**Affiliations:** 1 Department of Public Health, Section of Global Health, University of Copenhagen, CSS Campus, Building 9, Copenhagen, 1014 Denmark; 2 Gastrounit, Medical Section, Copenhagen University Hospital (Amager and Hvidovre Hospital), Hvidovre, Denmark; 3 Shree Hindu Mandal Hospital, Dar es Salaam, Tanzania; 4 School of Medicine, KCMC University, Moshi, Tanzania; 5 Monduli District Hospital, Monduli, Tanzania; 6 Department of Biomedical Sciences, University of Copenhagen, Copenhagen, Denmark; 7 Laboratory of Sports and Nutrition Research, Department of Human Physiology and Biochemistry, Riga Stradins University, Riga, Latvia; 8 Clinical Research Centre, Medical University of Bialystok, Bialystok, Poland

**Keywords:** Oral glucose tolerance test, Incretin hormones, Sex differences, Maasai, Sub-Saharan Africa

## Abstract

**Aims:**

Rural, agro-pastoralist Maasai in East Africa exhibit low prevalence of diabetes, yet little is known about their physiological response to glucose loads and whether sex has an impact on glucose metabolism, including incretin hormones.

**Methods:**

We included 58 (29 men, 29 women) adult Maasai without diabetes living in rural Tanzania. Clinical background characteristics were measured, and they were exposed to an oral glucose tolerance test (OGTT) after an overnight fast. Plasma glucose, insulin/C-peptide, glucagon, glucose-dependent insulinotropic polypeptide (GIP), and glucagon-like peptide-1 (GLP-1) were analysed.

**Results:**

Mean age was 34.8 (range 17–65) years, and mean body mass index (BMI) was 20.3 (range 14.0-30.9) kg/m^2^ with two individuals being overweight and 14 being underweight. Men had a higher mean fasting glucose concentration (5.2 vs. 4.9 mmol/L, *p* = 0.031), while women exhibited a higher OGTT-derived mean GLP-1 concentration at 2-h (8.5 vs. 5.1 pmol/L, *p* = 0.005). Total area-under-the-curve (tAUC) for GLP-1 was higher in women compared to men (1336 vs. 870 pmol x min, *p* = 0.011). Sex- and BMI-adjusted regression analyses for tAUC showed higher values of insulin, C-peptide and GLP-1 in women (*p* < 0.10).

**Conclusions:**

Sex differences were found in fasting glucose and OGTT-derived insulin/C-peptide and GLP-1 concentrations. Research using more sophisticated methodology is needed to further explain the glucose metabolism phenotype in Maasai people.

**Supplementary Information:**

The online version contains supplementary material available at 10.1007/s00592-026-02710-8.

## Introduction

Overweight and obesity, including abdominal obesity, are not uncommon in the Maasai [1], an agro-pastoralist people living in Kenya and Tanzania numbering approximately 1.6 mill [2, 3]. whose cardiometabolic health has been studied frequently for several decades [4, 5] In rural and urban Kenyan Maasai, glucose intolerance was primarily associated with increasing visceral adipose tissue in men but increasing body mass index (BMI (kg/m^2^) in women [6]. In rural, Kenyan Maasai, the detrimental effects of abdominal obesity have also been shown as higher insulin resistance, higher first-phase insulin secretion, but lower insulin action compared to other ethnic groups in rural Kenya [7]. Despite this, we observed low prevalence of diabetes mellitus (DM) of < 2.0% in rural Maasai with an equal distribution between men and women [6, 7]. These results stand in stark contrast to other indigenous populations ≥ 20 years of age such as the Native Americans in the USA and Aboriginal Australians with prevalence rates of ~ 15% and ~ 29%, respectively, and with substantial sex difference in prevalence rate in the latter population [8, 9].

Several studies have investigated sex differences in glucose metabolism in different populations from Denmark, Australia, Mauritius, and Japan, respectively [10–13], and these have shown that men tend to have a higher fasting plasma glucose while women tend to have higher 2-hour plasma glucose following an oral glucose tolerance test (OGTT). Thus, sex seems to play a role in the physiology of glucose metabolism.

Incretin hormones, i.e. glucose-dependent insulinotropic polypeptide (GIP) and glucagon-like peptide-1 (GLP-1), are important factors that stimulate nutrient-induced insulin secretion in response to meal intake, including oral glucose [14]. This phenomenon is known as the incretin effect, as oral glucose leads to the release of GIP and GLP-1 from specialized entero-endocrine cells in the gut – in contrast to intravenous glucose which bypasses the gut – with a subsequent blood glucose-lowering effect [15, 16]. Despite their central role in glucose metabolism, no information exists on incretin hormones in relation to oral glucose exposure based on studies in agro-pastoralist populations in Africa, including the Maasai people. Likewise, glucagon response in relation to glucose homeostasis has not been studied in the Maasai.

Thus, based on sex-specific differences in body composition, we aimed to study possible differences of the physiological response to an OGTT, with specific focus on incretin hormones in rural Maasai men and women without DM and with a wide range in age and BMI.

## Subjects, materials and methods

### Geographical area and participants

Data were collected in Monduli district of Arusha Region in North-Central Tanzania in June-August 2018 during the cold, dry season. Potential participants of Maasai origin from rural communities (collectively known as Monduli Juu) were informed about the study presented as a diabetes and cardiovascular disease project through a Maasai physician (JS). They were presented with details orally and in writing and signed a consent form or in case of illiteracy gave their thumbprint. Maasai ethnicity and age ≥ 15 years were inclusion criteria. Pregnancy, known diabetes or diabetes detected as part of the study, or severe chronic or infectious illness were exclusion criteria. To accommodate the study, a makeshift laboratory was set up at the Catholic primary School in Monduli Juu situated approximately 1700 m above sea level.

### Data collection

Each participant had to arrive at the study site after an overnight fast on two separate days within a week. A health assessment questionnaire for screening of diabetes and other illnesses (medical history), and pregnancy screening for the women using a human chorionic gonadotropin urine test (Quick-Strip Pregnancy test, Shanghai Eugene Biotech Co., Ltd, China) were performed.

On Day 1, each participant was measured in light clothing and barefooted, for height using a portable stadiometer (Charder HM200P Portstad, Taichung City, Taiwan), weight using a portable electronic scale (Tanita SC-330s, Tokyo, Japan), waist and hip circumference using a non-stretchable tape measure at the midpoint of the lower palpable rib and the iliac crest, and at the trochanter major, respectively. Mid-upper arm circumference (MUAC) was measured on the dominant arm using a specific MUAC tape measure (Raven Equipment, Dunmow, Essex, UK). BMI and waist-hip ratio were derived.

After receiving careful instructions on the procedure, each participant subsequently underwent an OGTT while seated. An antecubital vein cannula was inserted for blood sampling, and a fasting blood sample was collected, followed by ingestion of 75 g of glucose mixed in 300 mL of water within 5 min after the fasting blood sample. Collection of 10–12 mL of blood was done at time points 0, 10, 30, 60, 90 and 120 min. The samples were immediately stored on ice and centrifuged within 10 min. The plasma was separated and stored on ice until the end of the test day and then moved to a freezer (-20 °C) at Monduli District Hospital. Immediately following the end of the data collection period, the plasma samples were transferred to and stored at Kilimanjaro Clinical Research Institute in Moshi at -80 °C, until they were shipped to Denmark for analysis.

On Day 2, following an overnight fast, abdominal fat distribution, i.e. visceral adipose tissue (VAT) and subcutaneous adipose tissue (SAT) was measured using ultrasound-scanning technique (Aquila Basic Unit, Esaote, Pie Medical Equipment, Maastricht, the Netherlands) with a 3.5/5.0 MHz transducer (Probe Article no. 410638 Curved Array HiD probe R40 Pie Medical Equipment, Maastricht, the Netherlands). The measurements were carried out according to the protocol validated by [17]. Blood pressure was measured using an automatic M10-IT brachial blood pressure monitor (Omron, Kyoto, Japan) with the participant sitting in a chair with the cuff placed on the left arm. Three measures were taken with 2 min intervals with the first measure discarded, and an average of the subsequent two measures was recorded.

Haemoglobin was measured using venous blood on a portable HemoCue^®^ Hb 201 + device (HemoCue AB, Ängelholm, Sweden).

Dietary intake for each participant was estimated based on two interactive 24-hour recall interviews developed to collect dietary information in rural populations living in low and middle-income countries [18].

As the last measurement, an 8-minute ramped (15–33 cycles) sub-maximal step test stepping up and down a 21.5 cm high step followed by a 2-minute post-test period of sitting on a chair, recording heart rate throughout the test and post-test periods was performed in order to estimate aerobic fitness using a heart rate monitor (Garmin HRM3SS, Olathe, KS, USA) [19].

### Laboratory analyses

Plasma concentrations of glucose, insulin and C-peptide were analysed according to the sandwich principle on commercially available analysis kits (COBAS 6000 analyser 501 C, Roche, Germany). Plasma concentrations of total GIP, GLP-1 and glucagon were measured using commercially available sandwich ELISA kits (cat no: 10-1258-01, 10-1278-01, and 10-1271-01, respectively) (Mercodia, Uppsala, Sweden).

### Calculations of AUC and insulin indices

Total area under the curve (tAUC) for the OGTT measurements for glucose, insulin, C-peptide, GIP, GLP-1and glucagon, respectively, was calculated with the use of the trapezoidal method [20]. Incremental AUC (iAUC) was calculated by subtracting the fasting plasma results to capture the effect of the OGTT stimulus [21]. Hepatic insulin resistance was assessed by using the homeostasis model analysis – insulin resistance (HOMA-IR) according to the formula by [22], and early phase insulin secretion was calculated from the first 30 min. of the iAUC. The disposition index (DI) was calculated as early-phase insulin secretion/HOMA-IR, a measure of the functionality of the pancreas [23]. The Matsuda index was calculated as an assessment of whole-body insulin sensitivity according to the formula by [24]. Insulin/glucagon ratio (IGR) as an indicator of metabolic state was based on tAUC, and early phase GIP and GLP-1 secretion was based on iAUC of the first 30 min.

### Cut-offs for anthropometric, haemodynamic and biochemical disorders

BMI categories of underweight, overweight and obesity were defined as < 18.5, ≥ 25.0, and ≥ 30.0 kg/m^2^, respectively [25]. Abdominal obesity was defined as a waist circumference of 94 cm and 80 cm for men and women, respectively, and as waist-hip ratio *(WHR)* of 0.90 and 0.85 for men and women, respectively [25]. Mid-upper arm circumference (MUAC) of < 24.0 cm was defined as underweight [26].

Anaemia was defined as blood haemoglobin < 13.3 g/dL for men and < 12.3 g/dL for women, including an altitude correction factor of + 0.3 g/dL to avoid overestimation of haemoglobin levels [27].

For hyperglycaemic values, impaired fasting glucose (IFG) was defined as 6.1 to 6.9 mmol/L for the plasma fasting value, impaired glucose tolerance (IGT) was defined as < 7.0 mmol/L for fasting plasma value and ≥ 7.8 to < 11.1 mmol/L for the 120 min. value, and DM was defined as ≥ 7.0 mmol/L for the fasting plasma value, or ≥ 11.1 mmol/L for the 120 min. value [28].

### Statistics

Continuous background variables are presented as mean (SD) and summary measures, AUCs and indices are presented as mean (SD), or if skewed, data were log-transformed to determine p-value and then calculated as geometric mean (95% CI). Sex differences between variables were determined by Student’s *t*-test. For an assessment of the role of exposure variables for the outcome differences of AUCs for men and women, multivariable linear regression analyses were conducted using sex, age and BMI as fixed independent variables. The impact of BMI as an exposure variable on the outcome variables (glucose, insulin, C-peptide, GIP, GLP-1, and glucagon) was greater than the impact of WC, *WHR*,* VAT*,* SAT or VAT/SAT ratio* and therefore chosen as the anthropometric exposure variable.

### Ethical considerations

Ethical approval was obtained locally from Tumaini University, Moshi (Certificate no. 507), and from the National Institute of Medical Research in Tanzania (Ref. no. NIMR/HQ/R.8a/Vol IX/2806). All procedures were conducted in accordance with the Declaration of Helsinki and participants were treated as prescribed in the Tanzanian national guidelines.

### Data and resources availability

The data obtained and analysed in the current study are available on reasonable request.

## Results

### Number of eligible study participants and background characteristics

In total, 74 Maasai volunteered to participate in the study. We excluded one with known DM, 5 due to pregnancy, and 10 (1 male, 9 females) due to missing a 2-h blood test value. Thus, 58 individuals (29 men, 29 women) completed the study. Mean age was 34.8 (SD 11.9) (range 17–65) years, and mean BMI was 20.3 (SD 2.9) (range 14.0-30.9) kg/m^2^. Background characteristics by sex are presented in Table 1.

**Table 1:** Background characteristics of Tanzanian Maasai women and men (*n* = 58)

Variables	Men (*n* = 29)		Women (*n* = 29)		*P*-value
	Mean^1^	SD	Mean^1^	SD	
Age (years)	31.7	7.1	37.8	14.8	0.049
Anthropometrics/body composition					
Height (cm)	171.4	6.2	159.3	5.5	< 0.001
Weight (kg)	60.4	10.4	50.9	1.3	< 0.001
Body mass index (kg/m^2^)	20.5	2.9	20.1	3.8	0.56
Waist circumference (cm)^2^	78.0	8.6	73.0	7.4	0.020
Hip circumference (cm)^2^	88.8	11.1	90.7	7.2	0.44
Waist-hip ratio^2^	0.89	0.25	0.80	0.05	0.047
Mid-upper arm circumference (cm)^3^	26.8	2.9	26.2	2.6	0.40
Visceral adipose tissue (cm)^4^	5.8	1.8	5.3	1.2	0.25
Abdominal subcutaneous adipose tissue (cm)^3^	0.8	0.3	1.5	0.5	< 0.001
Visceral/subcutaneous fat ratio^5^	7.4	6.5;8.4	3.7	3.3; 4.2	< 0.001
Haemodynamics/biochemistry					
Systolic blood pressure (mmHg)^3^	121	11	112	12	0.008
Diastolic blood pressure (mmHg)^3^	83	8	80	10	0.22
Sitting heart rate (bts/min)^3^	68	11	77	11	0.003
Haemoglobin (mmol/L)	10.2	0.9	8.9	0.7	< 0.001
Daily dietary intake^4^ and fitness					
Energy intake (kJ)	16,397	3451	13,111	3790	0.002
Carbohydrate intake (%)	60.7	11.5	64.7	9.7	0.14
Fat intake (%)	28.2	6.5	25.2	6.3	0.21
Protein intake (%)	11.2	3.6	10.6	2.4	0.56
Relative protein intake (g · day^− 1^ · kg bw^− 1^)	2.04	0.93	1.93	0.98	0.66
Estimated aerobic fitness (mlO_2_ · min^− 1^ · kg^− 1^)^6^	40.4	6.0	33.8	5.6	< 0.001

^1^Mean (SD) or geometric mean (95% CI); ^2^*n* = 57; ^3^*n* = 56; ^4^*n* = 47; ^5^*n* = 46 ^6^*n* = 44

### OGTT-derived plasma concentrations

**Fig. 1:**
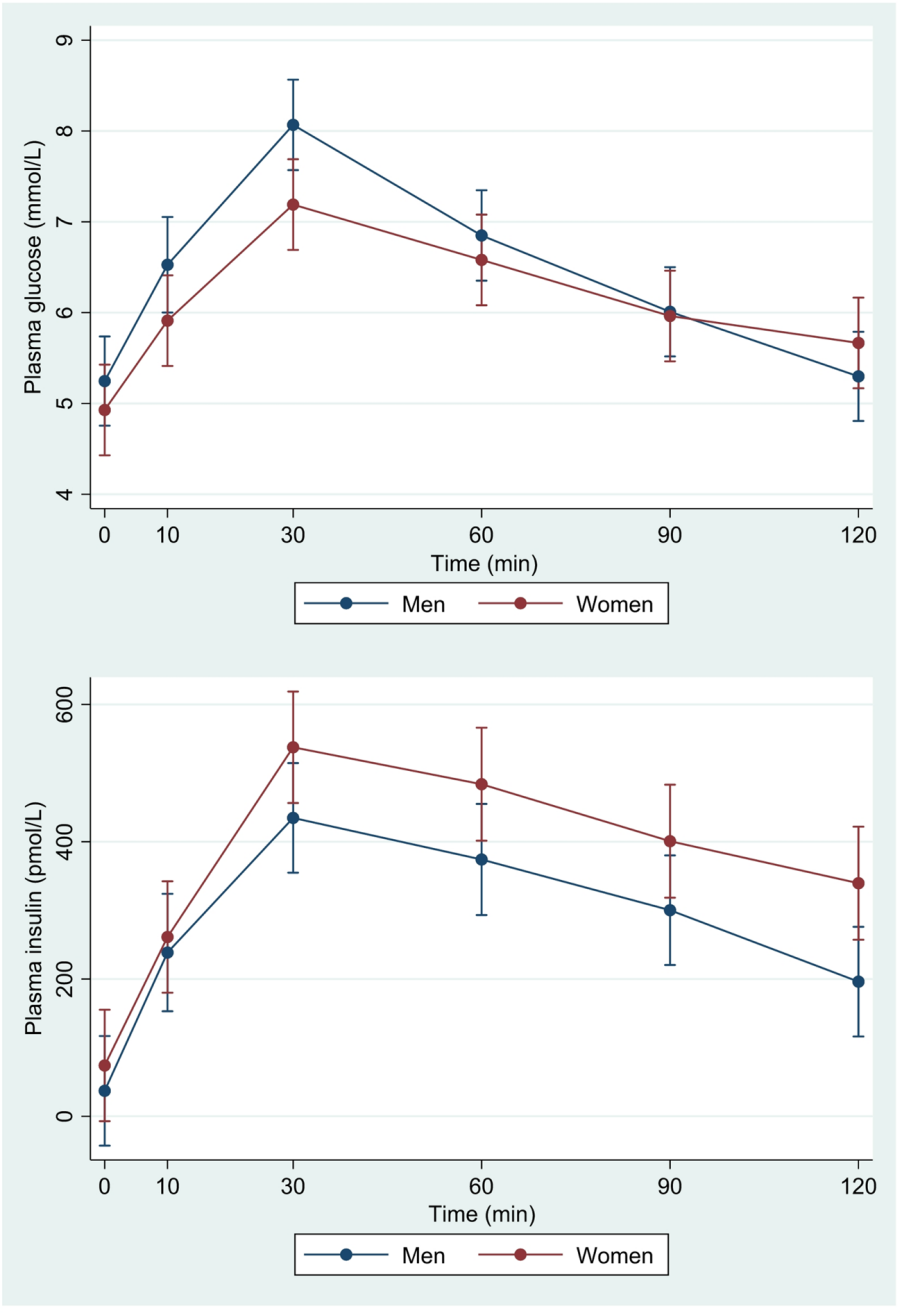

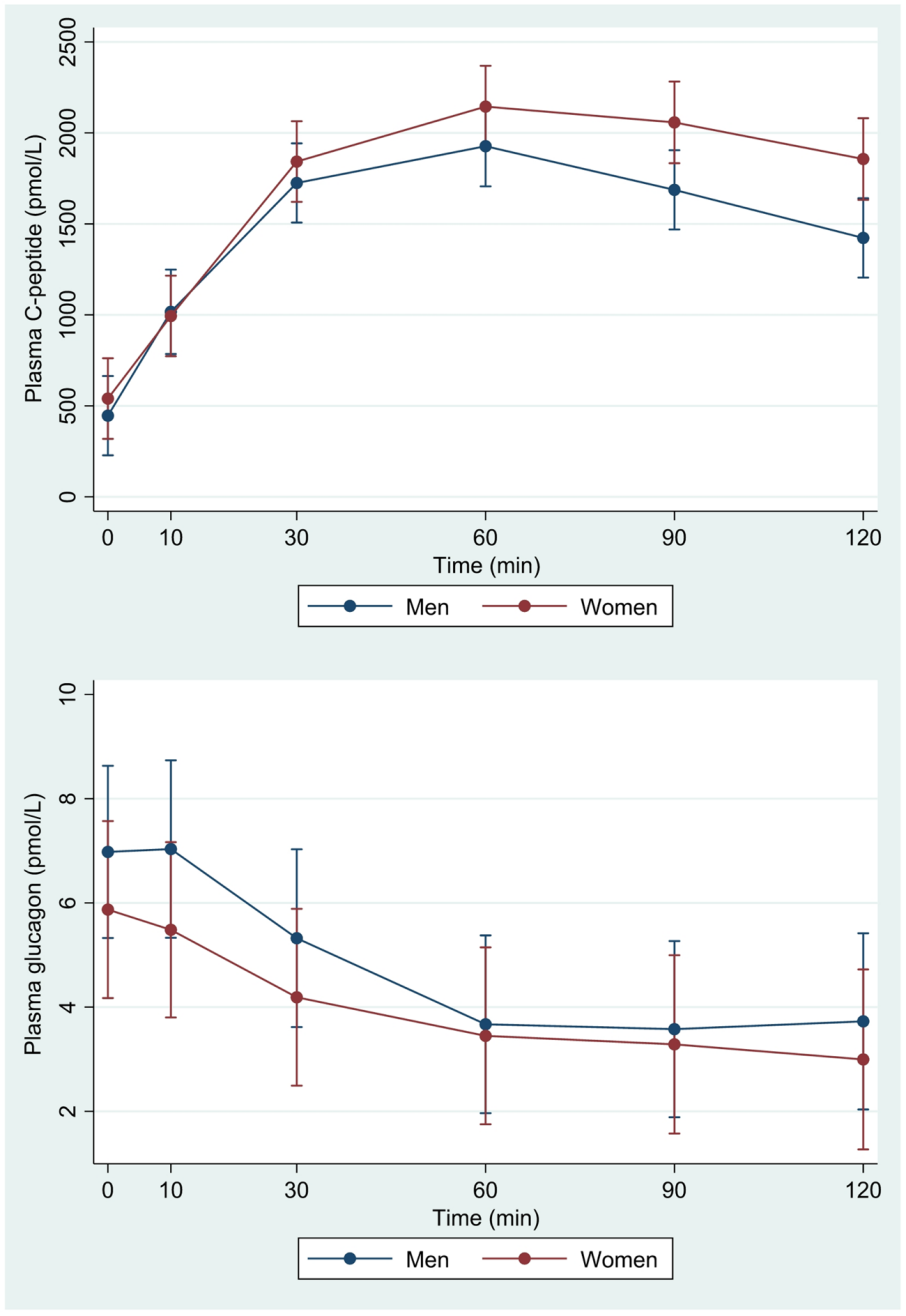

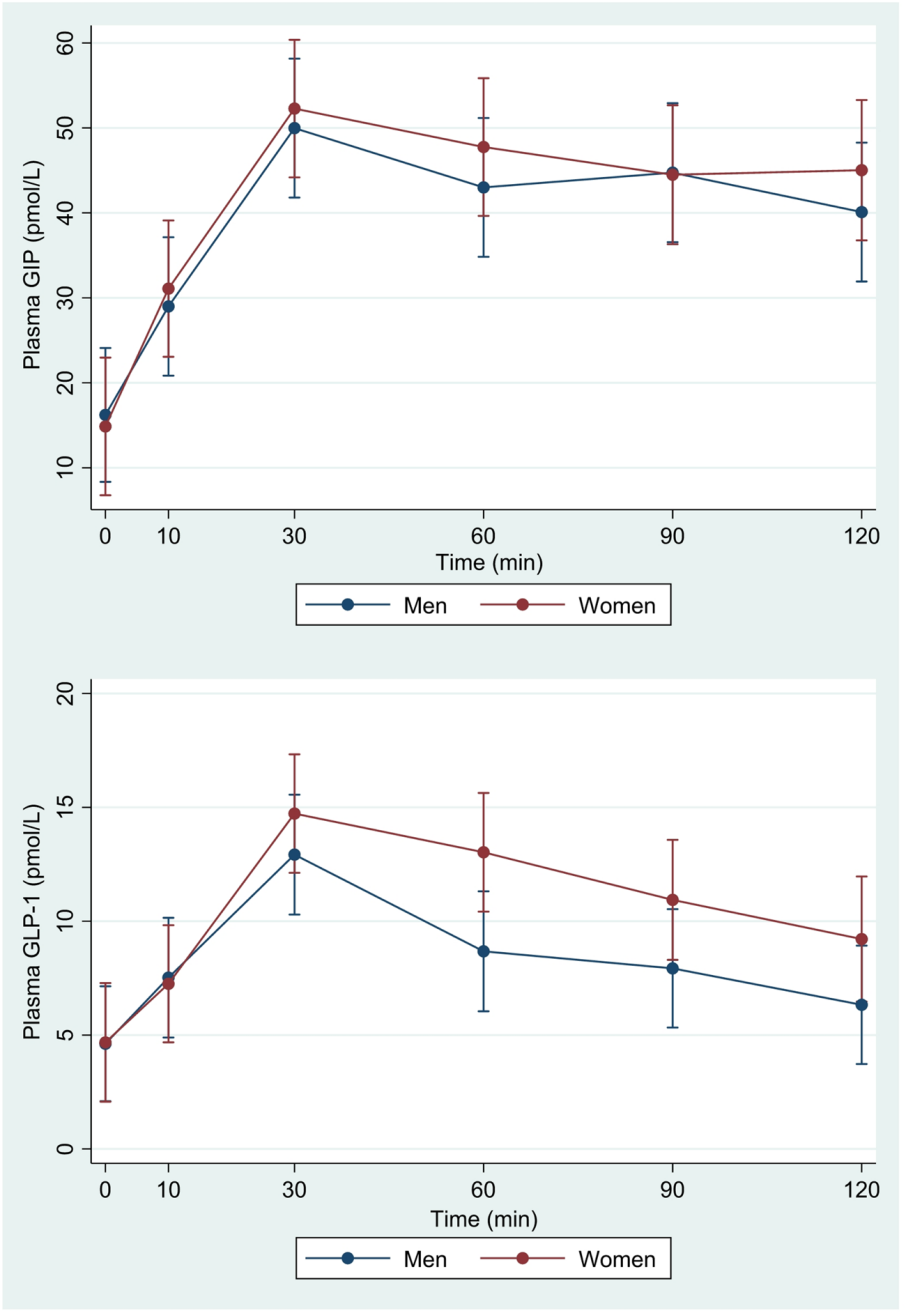
Associations between time (x-axis) and outcome variables (y-axis), i.e. total area-under-the-curve (units x min) following a standard 75 g oral glucose tolerance test in rural Maasai stratified by sex. GIP, glucose-dependent insulinotropic polypeptide and GLP-1, glucagon-like peptide-1. For all associations *n* = 58. Sex differences were only found in GLP-1 with women (1336 (95% CI: 1048; 1704) pmol/L x min) having higher concentrations compared to men (870 (95% CI: 690; 1097) pmol/L x min) (*p* = 0.011)

Based on the fasting test and OGTT, one male participant had IFG, and three participants (1 male, 2 females) had IGT, respectively. Fasting plasma glucose concentrations in men and women were significantly different at 5.2 (SD 0.6) vs. 4.9 (SD 0.5) mmol/L, respectively (*p* = 0.031). Based on the OGTT, there was a significant difference in geometric mean plasma GLP-1 with higher concentrations in women (8.5 (95%: 6.5; 11.0) pmol/L) than men (5.1 (95%:4.1; 6.5) pmol/L) (*p* = 0.005) at 120 min. For tAUC, GLP-1 was significantly higher in women (1336 (95% CI: 1048; 1704) pmol/L x min) compared to men (870 (95% CI: 690; 1097) pmol/L x min) (*p* = 0.011). All other tAUC comparisons were statistically non-significant. Comparisons by iAUC did not alter any of the results (data not shown). All results based on the OGTT are presented in Table 2, and tAUCs are graphically presented in Fig. 1.

**Table 2:** Fasting and 2-h glucose, insulin/C-peptide, glucagon and incretin hormones in Tanzanian Maasai (*n* = 58)

Variables	Men (*n* = 29)		Women (*n* = 29)		*P*-value
	Mean	SD/95% CI	Mean	SD/95% CI	
Simple summary measures					
Fasting glucose (mmol/L)	5.2	0.6	4.9	0.5	0.031
2-h glucose (mmol(L)^1^	5.1	4.6; 5.7	5.5	5.1; 6.1	0.22
Fasting insulin (pmol/L)^1^	51	42; 60	47	38; 57	0.54
2-h insulin (pmol/L)^1^	179	137; 234	255	197; 330	0.058
Fasting C-peptide (pmol/L)^1^	470	422; 523	472	423; 527	0.96
2-h C-peptide (pmol/L)^1^	1389	1217; 1585	1682	1453; 1948	0.051
Fasting glucagon (pmol/L)^1^	4.2	2.4; 7.4	4.1	3.0; 5.7	0.95
2-h glucagon (pmol/L)^1^	2.6	1.7; 4.1	2.0	1.2; 3.3	0.41
Fasting GIP (pmol/L)^1,2^	9.2	6.6; 12.8	11.1	8.0; 15.2	0.41
2-h GIP (pmol/L)^1,2^	35.5	29.5; 42.7	42.7	34.4; 53.0	0.19
Fasting GLP-1 (pmol/L)^1,3^	3.6	2.8; 4.5	3.9	3.0; 5.0	0.64
2-h GLP-1 (pmol/L)^1,3^	5.1	4.1; 6.5	8.5	6.5; 11.0	0.005
Area-under-the-curve (AUC)^1,4^					
Glucose AUC, total (mmol/L × min)	763	712; 817	744	698; 792	0.58
Insulin AUC, total (nmol/L × min)	37,739	33,166; 42,942	39,968	32,452; 49,225	0.65
C-peptide AUC, total (nmol/L × min)	183,097	165,294; 202,817	199,970	176,621; 226,406	0.27
Glucagon AUC, total (pmol/L × min)	466	348; 622	402	291; 553	0.48
GIP AUC, total (pmol/L × min)	4469	3795; 5263	5121	4166; 6294	0.29
GLP-1 AUC, total (pmol/L × min)	870	690; 1097	1336	1048; 1704	0.011
Indices					
HOMA-IR^1,5^	1.95	1.61; 2.36	1.69	1.36; 2.10	0.31
Early phase insulin secretion (pmol/L)^1,6^	7669	6027; 9758	7633	6225; 9361	0.98
Early phase C-peptide secretion (pmol/L)^1,7^	33,730	29,019; 39,207	32,794	28,949; 37,150	0.77
Disposition index^1,8^	3238	2406; 4357	3727	3050; 4556	0.41
Matsuda index^9^	16.2	6.2	18.1	7.8	0.34
Insulin/glucagon ratio^1,10^	76	55; 106	107	79; 146	0.12
Early phase GIP secretion (pmol/L × min)^1,11^	838	630; 1115	1061	826; 1364	0.20
Early phase GLP-1 secretion (pmol/L × min)^1,12^	217	163; 288	290	228; 369	0.11

^1^Geometric mean

^2^GIP: glucose-dependent insulinotropic polypeptide

^3^GLP-1: glucagón-like peptide-1

^4^Plasma concentrations over 120 min

^5^HOMA-IR: (insulin 0 min (µU/L) · glucose 0 min (nmol/L))/ 22.5 [22]

^6^Early phase insulin secretion: iAUC of first 30 min OGTT measurements

^7^Early phase C-peptide secretion: iAUC of first 30 min OGTT measurements

^8^Disposition index: Early phase insulin secretion/HOMA-IR [23]

^9^Matsuda index: 10,000 · (√(glucose 0 min · insulin 0 min · mean OGTT glucose · mean OGTT insulin))^−1^ [24]

^10^Insulin/glucagon ratio based on total AUC of OGTT-derived insulin and glucagon

^11^Early phase GIP secretion: iAUC of first 30 min OGTT measurements

^12^Early phase GLP-1 secretion: iAUC of first 30 min OGTT measurements

When tAUCs were adjusted for sex, age and BMI in regression analyses, borderline significant differences between men (reference group) and women were seen for insulin (11 724, 95% CI: -394; 23 841) nmol/L x min (*p* = 0.058), C-peptide (29 529, 95% CI: -4989; 64 047) nmol/L x min (*p* = 0.092), and for GLP-1 (464, 95% CI: -38; 966) pmol/L x min (*p* = 0.069), respectively. Results for glucose, GIP and glucagon were statistically insignificant (data not shown). Replacing BMI with WC, *WHR*,* VAT*,* SAT or VAT/SAT ratio* when adjusting the regression analyses of tAUCs did not alter the results (see Supplementary Table). Furthermore, omitting individuals having pre-DM (*n* = 4) from the adjusted regression analyses did not alter the results (data not shown).

### Under/overweight, hypertension and anaemia

Underweight was found in fourteen (6 males, 8 females) and eight (3 males, 5 females) participants by BMI and MUAC, respectively. Based on BMI, one male participant was overweight, and one male participant was obese. Abdominal obesity was found in five (2 males, 3 females) and one female participant by WC and WHR, respectively. Nine participants had hypertension (5 males, 4 females). One female participant had anaemia.

## Discussion

In this study, based on a standard 75 g OGTT, we demonstrated that in traditionally living Maasai, men had higher mean fasting plasma glucose compared to women, while there was a tendency towards higher mean 120 min. plasma insulin and C-peptide in women compared to men. At 120 min. of the OGTT, mean plasma GLP-1 was higher in women compared to men. When calculating AUC (total or incremental), women had higher mean plasma GLP-1 concentrations. Furthermore, adjusted AUC insulin, C-peptide and GLP-1 plasma levels were borderline significant, with higher values for women.

The higher fasting plasma glucose in Maasai men supports similar results in studies carried out in different population groups, i.e. Mauritius, Australia, Denmark, and Japan, respectively [10–13], even though we did not find higher fasting insulin concentrations or higher HOMA-IR in men. The latter is mainly an indication of hepatic insulin resistance [29]. Thus, hepatic insulin resistance did not seem to be affected by higher central fat accumulation (*WC*,* WHR*, and VAT/SAT ratio) among men in the current study population. Even though 120 min. glucose levels were not different between the sexes, there was a tendency towards higher 120 min. insulin and C-peptide concentrations in women. This was supported by AUC concentrations for insulin and C-peptide adjusted for sex and BMI, indicating that overall, during a standard OGTT and at the 120 min. time-point, higher insulin/C-peptide concentrations in women is a result of inter-sex physiological differences. Women seem to exhibit a greater insulin secretion capacity than men [30]. This is possibly due to differences in height and thereby lean body mass, i.e. there is a higher glucose-to-muscle mass ratio in women compared to men [31], and therefore an increased need for insulin in women. As there was no difference in early phase insulin or C-peptide secretion (first 30 min. of the OGTT) between men and women, the higher 120 min. insulin level could be a compensatory mechanism for a lower peripheral insulin sensitivity in women compared to men, which conflicts with previous studies showing higher peripheral insulin sensitivity in women, albeit under non-physiological conditions (clamp studies) due to enhanced glucose uptake by skeletal muscle in women [32, 33]. Nonetheless, our results of whole-body insulin sensitivity as calculated through the Matsuda index did not support sex differences.

The incretin hormones GIP and GLP-1 stimulate insulin secretion in a glucose-dependent manner [34, 35]. Therefore, enhanced OGTT-derived insulin concentration, which we observed in women, may also reflect sex differences in GIP and GLP-1 secretion. While we found no differences in GIP secretion, plasma concentrations of GLP-1 at 120 min. and tAUC GLP-1 were higher in women. Sex differences in incretin hormone response during the 75 g OGTT between men and women have previously been reported in individuals with normal glucose tolerance as well as hyperglycaemia, albeit with different results [36, 37]. In a twin study discordant for type 2 diabetes including obese Danish individuals with an average age of 60 years, the authors showed that plasma GIP and GLP-1 concentrations were higher in women following a 180 min. OGTT [36], while young Japanese individuals with normoglycaemia (average age 25 years) exhibited higher GIP concentrations among men following a 120 min OGTT [37]. The discrepancy between the incretin response to an OGTT, including the current findings, could be explained by differences in age, ethnicity and body composition. Furthermore, in an epidemiological study including a sub-group of 774 Danish individuals with normoglycaemia, women had substantially higher iAUC at 30 and 120 min. than men when adjusted for age and BMI [38].

Plausible physiological explanations for the higher GLP-1 plasma concentrations in women could be differences in gastric emptying and/or caused by sex-specific hormones. As for the former, little information is available in normoglycaemic individuals, and the available evidence shows conflicting results in difference of gastric emptying rate between men and women [39, 40]. Sex hormones, on the other hand, have been shown to play a role in relation to not only GLP-1 secretion, but also insulin and glucagon secretion. Estrogens act on the pancreas to increase insulin secretion, while they decrease glucagon secretion [39, 40]. Furthermore, enteroendocrine L-cells express receptors for estrogen and for progesterone, and they secrete more GLP-1 in response to these two steroids [41].

It is of note that Nielsen et al. [41]. recently reported a similar OGTT-derived incretin effect in Africans from Tanzania comparing groups of individuals with DM (diagnosed from OGTT or HbA1c) or without DM. The authors speculate that the lack of difference could be due to the relatively young age of the participants (average age < 50 years) and therefore possibly at an early stage in their diabetes disease. However, a difference in the assays utilized compared to the current study could also explain the “negative” finding in the study by Nielsen et al. [41].

When added together, the favourable incretin profile in women (higher 2-h as well as tAUC GLP-1 concentrations) together with increased insulin/C-peptide concentrations (borderline significant) do not result in a lower plasma glucose tAUC compared to men. This could be due to the fact that by giving the same glucose load to men and women, the relative glucose load was higher in women due to their lower weight (~ 10 kg difference), and thereby lower lean body mass. Thus, women are better at handling the blood glucose, probably as a consequence of higher GLP-1 concentrations.

There is solid evidence that the composition of dietary intake has an impact on glucose homeostasis, including postprandial and glucose challenge tests [42]. In the current study, the composition of the habitual dietary intake (average of two 24-h recall interviews) at the macronutrient level was similar among Maasai men and women, including very high relative protein intake in both sexes (~ 2 g · day^− 1^ · kg bw^− 1^). Aerobic fitness – a proxy for physical activity – is also related to glucose homeostasis and insulin sensitivity, and the average fitness levels (~ 16% higher in men) were moderate for both sexes and within normal level of difference between men and women, i.e. ~15–25% for this parameter [43, 44].

### Limitations of the study

The study had several limitations, which could have affected the results of the OGTT. Apart from securing a state of overnight fasting, we could not secure standardization of the dietary intake for 3 days prior to the OGTT as recommended. Furthermore, we had to allow the study participants to walk slowly to a nearby restroom during the OGTT if requested. Regarding insulin indices, we acknowledge that HOMA-IR as well as the Matsuda index are indirect surrogates derived from the OGTT rather than clamp-based measures. Due to the “field conditions”, we were only able to collect simple anthropometric and estimated aerobic fitness data. Thus, neither body composition nor fitness could be measured with high precision. Finally, the results presented should be interpreted with caution and need replication in other independent datasets among Maasai or other agro-pastoralist groups with similar lifestyle and -conditions.

In conclusion, in rural Maasai men and women of wide ranges of age (17–65 years) and BMI (14.0–30.9 kg/m^2^) and without having DM, fasting and OGTT-derived plasma glucose concentrations showed a higher average fasting concentration in men, while women exhibited higher average 120 min. insulin, 120 min. GLP-1 and tAUC GLP-1 concentrations. A larger sample size and inclusion of more sophisticated methodologies should be included in future studies to investigate sex-specific biology in this unique population known to have a low prevalence of DM despite growing obesity at the population level.

## Supplementary Information

Below is the link to the electronic supplementary material.

Supplementary Material 1
