# Supplementary material for: Differences in physiological response to an oral glucose tolerance test in adult Maasai men and women
Source: Acta Diabetol. 2026 May 22;63(8):1363–73. doi: 10.1007/s00592-026-02710-8 (PMC13468978; doi:10.1007/s00592-026-02710-8)
Supplement: Supplementary file 1 [file 592_2026_2710_MOESM1_ESM.docx]

| **tAUC variables^4^** | ***B*-coefficient** | **95% CI** | **P-value** | | |
| --- | --- | --- | --- | --- | --- |
| ***Adjusted for sex, age, BMI***^5^ |  |  |  | | |
| Glucose (mmol/L x min) | -26 | -100; 48 | 0.48 | | |
| Insulin (pmol/L x min) | 11724 | -394; 23841 | 0.058 | | |
| C-peptide (pmol/L x min) | 29529 | -4989; 64047 | 0.092 | | |
| Glucagon (pmol/L x min) | -68 | -318; 183 | 0.59 | | |
| GIP (pmol/L x min)^6^ | 793 | -514; 2101 | 0.23 | | |
| GLP-1 (pmol/L x min)^7^ | 448 | -37; 933 | 0.069 | | |
| ***Adjusted for sex, age, WC***^8^ |  |  |  | | |
| Glucose (mmol/L x min) | -25 | -2; 5 | 0.80 | | |
| Insulin (pmol/L x min) | 14060 | 984; 27137 | 0.036 | | |
| C-peptide (pmol/L x min) | 35440 | -1619; 72499 | 0.060 | | |
| Glucagon (pmol/L x min) | -95 | -350; 159 | 0.45 | | |
| GIP (pmol/L x min)^6^ | 252 | -1118; 1622 | 0.37 | | |
| GLP-1(pmol/L x min)^7^ | 315 | -157; 787 | 0.19 | | |
| ***Adjusted for sex, age, WHR****^9^* |  |  |  | | |
| Glucose (mmol/L x min) | -18 | -97; 61 | 0.65 | | |
| Insulin (pmol/L x min) | 11827 | -1247; 24901 | 0.075 | | |
| C-peptide (pmol/L x min) | 29929 | -6970; 66829 | 0.11 | | |
| Glucagon (pmol/L x min) | -86 | -337; 164 | 0.49 | | |
| GIP (pmol/L x min)^6^ | 580 | -840; 2001 | 0.42 | | |
| GLP-1(pmol/L x min)^7^ | 353 | -116; 823 | 0.14 | | |
| ***Adjusted for sex, age, VAT***^10^ |  |  |  | | |
| Glucose (mmol/L x min) | -20 | -108; 68 | 0.65 | | |
| Insulin (nmol/L x min) | 12768 | -1780; 27317 | 0.084 | | |
| C-peptide (pmol/L x min) | 33078 | -7214; 73471 | 0.11 | | |
| Glucagon (pmol/L x min) | -46 | -326; 3 | 0.74 | | |
| GIP (pmol/L x min)^6^ | 828 | -691; 2347 | 0.28 | | |
| GLP-1 (pmol/L x min)^7^ | 398 | -155; 951 | 0.15 | | |
| ***Adjusted for sex, age, SAT***^11^ |  |  |  | | |
| Glucose (mmol/L x min) | -25 | -120; 71 | 0.61 | | |
| Insulin (pmol/L x min) | 1173 | -13375; 15722 | 0.87 | | |
| C-peptide (pmol/L x min) | 7548 | -34780; 49877 | 0.72 | | |
| Glucagon (pmol/L x min) | -140 | -445; 166 | 0.36 | | |
| GIP (pmol/L x min)^6^ | 1532 | -123; 3187 | 0.069 | | |
| GLP-1 (pmol/L x min)^7^ | 480 | -116; 1077 | 0.11 | | |
| ***Adjusted for sex, age VAT/SAT***^12^ |  |  |  | | |
| Glucose (mmol/L x min) | -27 | -163; 110 | 0.69 | | |
| Insulin (pmol/L x min) | 19 | -22426; 22464 | 1.0 | | |
| C-peptide (pmol/L x min) | 20492 | -43079; 84063 | 0.52 | | |
| Glucagon (pmol/L x min) | -158 | -582; 266 | 0.45 | | |
| GIP (pmol/L x min)^6^ | 1923 | -398; 4244 | 0.10 | | |
| GLP-1 (pmol/L x min)^7^ | 373 | -453; 1199 | 0.37 | | |
|  |  |  |  | | |

**Supplemental Table.** Adjusted regression analyses^1^ of OGTT^2^-derived tAUC^3^ in relation to glucose, insulin, C-peptide, glucagon and incretin hormones in Tanzanian Maasai (n=58)

^1^Women compared to men; ^2^OGTT, oral glucose tolerance test; ^3^tAUC, total area-under-the-curve; ^4^Plasma concentrations over 120minutes; ^5^BMI: body mass index; ^6^GIP: glucose-dependent insulinotropic polypeptide;^7^GLP-1: glucagón-like peptide-1; ^8^WC, waist circumference; ^9^WHR, waist-hip ratio; ^10^VAT, visceral adipose tissue; ^11^SAT, subcutaneous adipose tissue; ^12^VAT/SAT, visceral/subcutaneous adipose tissue ratio
